# Effectiveness of a guided internet-based intervention in reducing procrastination among university students – a randomized controlled trial

**DOI:** 10.1016/j.invent.2025.100878

**Published:** 2025-09-29

**Authors:** Arpana Amarnath, Sevin Ozmen, Chris van Klaveren, Annemieke van Straten, Julia Pei, Leonore de Wit, Rasmus E. Raabe, Caring Universities Consortium, Pim Cuijpers, Sascha Y. Struijs

**Affiliations:** a Department of Clinical, Neuro and Developmental Psychology, Amsterdam Public Health research institute, Vrije Universiteit Amsterdam, the Netherlands; b Department of Psychiatry, Faculty of Medicine, University of British Columbia, Vancouver, BC, Canada; c WHO Collaborating Centre for Research and Dissemination of Psychological Interventions, the Netherlands; d Institute of Clinical Psychology, Leiden University, Leiden, the Netherlands; e Amsterdam Public Health Research Institute, Amsterdam, the Netherlands; f Amsterdam Center for Learning Analytics, Vrije Universiteit Amsterdam, Amsterdam, the Netherlands

**Keywords:** Internet-based intervention, Guided intervention, Procrastination, University students, Mental heath

## Abstract

Procrastination is highly prevalent among students and has several negative consequences, affecting academic performance, mental health, and prospects for future professional development. However, there exists a treatment gap, with there being many more students with problems than those receiving help. This study aims to assess the effectiveness of a guided internet-based intervention, *GetStarted*, in addressing procrastination among college students. In this two-arm randomized controlled trial, 403 students were randomly assigned to *GetStarted* or waitlist control. The primary outcome was the difference in self-reported procrastination behaviours between intervention and control measured on the Irrational Procrastination scale (IPS) at post-test (4 weeks post-baseline). In addition, long-term effects based on the difference in the IPS scores from baseline to 6-months follow-up were assessed in the intervention group. Secondary outcomes were differences in depressive symptoms, anxiety, stress, and mental health-related quality of life. All analyses were based on the intent to treat principle. The Random Forest Lee bounds approach was applied as a sensitivity and robustness analysis. The sociodemographic characteristics of the participants were examined as treatment moderators. Finally, treatment acceptability was assessed through satisfaction with treatment, program usability, satisfaction with e-coach, and treatment adherence. Our results revealed that *GetStarted* was significantly effective in reducing procrastination at the post-test (Cohen's *d =* 0.40), and this effect remained stable at 6-month follow-up (*p* < .001). The intervention group also experienced reductions in depressive symptoms, anxiety, and stress, along with an increase in quality of life from baseline to post-test and 6-month follow-up, although these changes were not statistically significant apart from perceived stress. No significant moderators influenced treatment effectiveness. Overall, participants reported good acceptability of the treatment. *GetStarted* offers an effective, flexible, and low-intensity solution for treating procrastination, with the potential to prevent common mental health issues among college students.

**Trial registration:**

This trial is registered at ClinicalTrials.gov Protocol Registration and Results System (Trial number: NCT05478096).

## Highlights

• Procrastination is common in university students and is associated with several negative consequences
• Guided online interventions may overcome barriers to treatment, but evidence remains limited and methodologically weak
• *GetStarted* is an effective, flexible and low intensity program that reduces procrastination up to 6 months
• No significant secondary effects were found for depressive symptoms, anxiety and quality of life
• Students reported high satisfaction with both the intervention and their e-coaches

## 1 Introduction

Procrastination is a widespread problem among students. Procrastination refers to intentionally delaying the completion of necessary tasks (Steel, 2007). Approximately 70–90 % of students identify as procrastinators worldwide (Sederlund et al., 2020), with over 50 % procrastinating consistently and problematically (Wilson and Nguyen, 2012; Solomon and Rothblum, 1984; Tice and Baumeister, 1997; Pychyl et al., 2000; Schouwenburg et al., 2004; Steel, 2007; Ozer et al., 2009). In addition, over half of the students who identify their procrastination as problematic indicate that they would like to address and treat this problem (Grunschel and Schopenhauer, 2015). In an academic context, several studies have indicated that procrastination negatively affects students' academic progress and well-being (Kim and Seo, 2015; Sirois and Pychyl, 2016; Hayat et al., 2020). This includes academic failure, stress, anxiety, depression, and lower prospects for future professional development. In addition, when left unaddressed, procrastination can lead to increased societal and economic costs associated with mental health concerns and errors associated with rushing (Wilson and Nguyen, 2012). Given the range of possible adverse effects of procrastination on students, developing effective treatments and offering support wherever needed is essential.

Interventions with study-related targets such as procrastination and time management are more accepted by university students than interventions targeting specific disorders (Kählke et al., 2024). However, there exists a treatment gap, with there being many more students with problems than those receiving help. For instance, there has been an increase in demand for counselling services in universities and a strain on resources, resulting in a lack of specialized programs such as one for procrastination (Gallagher, 2015). This problem remains relevant, with only a few studies evaluating the effectiveness of interventions targeting procrastination (Van Eerde and Klingsieck, 2018; Rozental et al., 2018b; Turner and Hodis, 2023). In addition, treating procrastination, specifically in a student population, is difficult. The first and most significant barrier is identifying students who struggle with procrastination. While most students who procrastinate tend to identify their behaviours as problematic and would like to overcome their procrastination tendencies (Steel, 2007), not all seek help due to the self-regulatory problems inherent in procrastination (Giguère et al., 2016; Åsberg et al., 2024). The second barrier includes a range of attitudinal and practical factors that further reduce help-seeking behaviours, specifically among students. Research has pointed out that students prefer to solve their problems themselves, possess a limited sense of necessity for assistance, exhibit sensitivity to the negative connotations linked with mental well-being and seeking treatment, face constraints in terms of time and financial means for therapy, and typically possess inadequate awareness regarding the support options accessible to them (Czyz et al., 2013; Dunley and Papadopoulos, 2019; Eisenberg et al., 2007). The third barrier is treatment adherence. Even when students do seek help, sustained engagement with the treatment process remains a challenge. In line with this, Rozental et al. (2015b) found that many individuals undergoing procrastination treatment struggle to maintain a consistent commitment to the treatment process. Therefore, developing and delivering a treatment that can overcome several or most of these barriers is important. The current study was designed with these barriers in mind, aiming to evaluate an intervention that is accessible, low-cost, acceptable to students, and capable of supporting adherence even among those who typically struggle to stay engaged.

Internet-based interventions, characterized by its easy accessibility, anonymous nature, and adaptable scheduling, could offer an economically viable solution to several of the barriers mentioned above (Carlbring et al., 2023; Andersson et al., 2014; Ebert et al., 2017; Cuijpers et al., 2019b). Previous studies evaluating the effectiveness of e-health interventions to reduce procrastination have shown promising results (Eckert et al., 2018; Lukas and Berking, 2018). For instance, chat-based counselling was as effective as face-to-face counselling in decreasing procrastination behaviours (Gieselmann and Pietrowsky, 2016). Similarly, Rozental et al. (2018a) reported that unguided internet-based treatments were as effective as group-based interventions for managing procrastination. However, these studies often lack an inactive control group, making it difficult to isolate the precise impact of these interventions. In addition, studies with a comparison to an inactive control either do not focus on the student population or suffer from limitations such as small sample sizes and a high risk of bias, particularly in blinding outcome assessors (Rozental et al., 2018b). To address these limitations, the present trial includes a waitlist control group and is adequately powered to detect small-medium treatment effects in a student sample.

Another important consideration is that of guidance. A recent single-session intervention study for procrastination indicated that their participants (who were all students) expressed difficulties in adopting treatment feedback and advice without support (Åsberg et al., 2024). While guided treatments tend to have better treatment outcomes, unguided treatments are more scalable and affordable (Cuijpers et al., 2019c; Karyotaki et al., 2017). A promising middle ground involves guidance delivered by non-clinicians which is found to be as effective as clinician guidance (Leung et al., 2022).In the current study guidance was delivered by clinicians in training, specifically master and 3rd year bachelor clinical psychology students, who fall within the broader category of non-clinicians, yet possess formal psychological training and operate under structured supervision. Prior research has shown that guidance from psychology students can be both feasible and effective in digital interventions (Wang et al., 2017). This model combines the scalability and cost-efficiency of non-clinical support with a higher degree of quality assurance, and helps address barriers linked to the limited availability of licensed professionals (Watkins et al., 2012).

Therefore, the primary objective of this study is to assess the effectiveness of a guided internet-based intervention, *GetStarted*, in addressing procrastination among university students. In this trial, procrastination was assessed using a validated self-report measure that captures the participants' habitual tendency to engage in irrational, self-defeating procrastination, and we refer to this outcome as procrastination henceforth. Given the known associations between procrastination and various psychological factors (Sirois, 2007; Flett et al., 2016; Stead et al., 2010; Beutel et al., 2016; Rozental et al., 2022), we will also examine the impact of this intervention on secondary outcomes such as depression, anxiety, stress, and mental health related quality of life. Moreover, we intend to explore potential factors that may influence the effectiveness of the treatment, including socio-demographic characteristics of the participants, satisfaction levels with the treatment and e-coach, system usability, and adherence to the treatment protocol. This investigation aims to pinpoint specific demographic groups that could derive the greatest benefit from the intervention. While previous research has demonstrated the potential of internet-based interventions for procrastination, few studies have tested guided programs tailored for university students, using rigorous methodology, and exploring long-term and secondary mental health outcomes. This study helps fill that gap by evaluating *GetStarted*, designed collaboratively with students and delivered with structured guidance.

## 2 Methods

### 2.1 Study design/setting

A two-armed, randomized controlled trial comparing the effectiveness of a guided internet-based intervention program, *GetStarted*, to a waitlist control group receiving no intervention for four weeks. A waitlist control condition was chosen because this trial represented an early-stage evaluation of *GetStarted*, aimed at assessing its initial efficacy in a real-world student setting while also ensuring ethical access to the intervention for all participants. This study is conducted within the Caring Universities project. Caring Universities is a consortium of 7 universities in the Netherlands. The project is a part of the World Health Organization (WHO) World Mental Health International College Student Initiative (WMH-ICS; Cuijpers et al., 2019a), aimed at improving our knowledge of university students' mental well-being and subsequently offering accessible web-based interventions to students in need.

Participants were randomized to either the intervention or control group using a computer random sequence generator. Treatment assignment was unblinded because a waitlist control condition was used. This study was approved by the Scientific and Ethical Review Board of all participating universities in the Netherlands: Vrije University Amsterdam, Leiden University, Maastricht University, and Utrecht University, Erasmus University, University of Amsterdam and Inholland University of Applied Sciences. The trial is registered on ClinicalTrials.gov (NCT05478096) and is conducted and reported according to the CONSORT 2010 statement and the guidelines for executing and reporting research on internet-based interventions (Schulz et al., 2010; Proudfoot et al., 2011). The program (*GetStarted*) is administered at all the participating universities mentioned above.

### 2.2 Participants and procedure

Participants were students enrolled at one of the participating universities of the Caring Universities consortium. They were recruited to the trial between November 2021 and October 2022 via flyers, posters, advertisements, and through the staff who work directly with students such as the student psychologists, study advisors, and lecturers. Students were eligible if they were above the age of 16, had access to a laptop/computer or mobile device with internet access, scored above 32 on the Irrational Procrastination Scale (Rozental et al., 2015b) and provided informed consent. Details on participant recruitment, sample size estimation, randomization, and study procedures are reported in the published protocol (Amarnath et al., 2023).

All assessments were conducted through the Caring Universities platform. The baseline assessment was administered immediately before participants began module 1 (week 0), followed by assessments at post-test (4 weeks post baseline), and 6-month follow-up. Participants were instructed to complete one module per week, starting with module 1 in week 0, allowing all five modules to be completed within 4 weeks. At each time point, participants received a questionnaire link, with reminder emails sent at 1 and 2 weeks, and follow-up phone calls if needed. More details of outcome assessment time points are reported in the protocol paper (Amarnath et al., 2023).”

### 2.3 Intervention and control condition

Participants allocated to the intervention group received the *GetStarted* intervention. This intervention was developed based on the principles of cognitive behavioural therapy. It consisted of five primary modules and four optional modules, all accessible via internet-enabled devices such as computers, laptops, tablets, and mobile phones. Each module required approximately 30–40 min to complete, and participants were encouraged to complete at least one module per week. Participants could access the intervention through the Caring Universities platform (platform.caring-universities.com) via a unique username and password. They also had the option to access the intervention anonymously. Participants who remained inactive for two weeks without completing a module received automated email reminders. *GetStarted* has previously shown to be acceptable and feasible for college students (Ozmen et al., 2025).

The five main modules were centred on the psychological mechanisms that commonly underlie procrastination, drawing on principles from cognitive behavioural therapy (CBT). These include (1) psychoeducation on the process of procrastination, (2) journaling to understand the individual psychological mechanisms underlying procrastination, (3) unravelling cognitive distortions that lead to procrastination, (4) cognitive restructuring, and (5) relapse prevention. These modules were considered foundational and relevant for all students struggling with procrastination.

The four optional modules became available after the participants completed the second core module. These modules provided practical tools such as time management, task decomposition, strategic planning, and motivational techniques. Since these modules may not be relevant to all participants, they were designed to be self-selected based on individual needs.

All nine modules encompassed psychoeducation, reflective questioning, interactive exercises, and assignments. The program's content was delivered in both textual and visual formats, incorporating images, infographics, and videos. More detailed information on the program content is available in the published protocol (Amarnath et al., 2023) and the feasibility trial paper of *GetStarted* (Ozmen et al., 2025).

Participants allocated to the waitlist control group did not receive any intervention for four weeks following randomization. However, they were at liberty to use customary treatment alternatives, such as consulting general practitioners, student psychologists, and academic counsellors, as they saw fit. After the 4-week waiting period, students were invited to use *GetStarted.*

### 2.4 Guidance and intervention integrity

During the registration process, participants were given the option to participate anonymously and were presented with a random selection of three available e-coaches. They could choose one of these e-coaches to support them throughout the program based on their photo and a brief bio, enabling them to avoid any coach they might know personally. These e-coaches offered asynchronous written feedback throughout the platform within 72 h (weekdays only) following the completion of each module by the participant. The primary objective was to offer support, enhance motivation, and encourage adherence.

The e-coaches were master students and 3rd year bachelor students in clinical psychology. They had specialized theoretical and skill-based knowledge of low-intensity treatments. Before commencing their coaching responsibilities, they underwent 6 h of comprehensive training encompassing their role as an e-coach, analyses of written language, determination of core messages, motivational interviewing training, effective feedback structuring, crisis management, protocol adherence, and several practical training sessions. They also attended weekly one-hour intervision meetings which were supervised by the research team.

### 2.5 Safety monitoring

Given the low-risk nature of internet-based CBT (Karyotaki et al., 2018b), no standardized adverse event checklist was used. However, participants were monitored by the trained e-coaches who were instructed to report any suspected crisis situations (e.g., suicidal ideation) to the supervisory team. In such cases, participants were contacted by a qualified mental health professional and referred to external care if necessary.

## 3 Measures

### 3.1 Primary outcome

The Irrational Procrastination Scale (IPS) was used to evaluate participants' procrastination tendencies by assessing the extent to which they procrastinated (Steel, 2002, 2010). The IPS was chosen because it is brief, accessible and one of the most widely used instruments to assess procrastination in student populations. This scale consists of nine items scored on a five-point Likert scale ranging from 1 (very seldom or not true to me) to 5 (very often true or true to me). Possible scores range from 9 to 45, with higher scores indicating more pronounced procrastination tendencies. A cut-off score of 32 was used to identify those with problematic procrastination (Rozental et al., 2015b). The IPS has demonstrated strong internal consistency, validity, and a robust-test-retest reliability (Steel, 2010; Guilera et al., 2018; Rozental et al., 2014; Shaw and Zhang, 2021). For this study, the IPS demonstrated an acceptable internal reliability, with a Cronbach's α of 0.74.

### 3.2 Secondary outcomes

The secondary outcomes were symptoms of depression, anxiety, stress, and quality of life.

The Patient Health Questionnaire (PHQ-9; Kroenke and Spitzer, 2002) was used to assess depressive symptoms. A commonly used and accepted instrument for depressive symptoms, (Kroenke et al., 2010; Wittkampf et al., 2007), the PHQ-9 consists of nine items scored on a four-point Likert scale ranging from 0 (not at all) to 3 (nearly every day). Possible scores range from 0 to 27 with higher scores indicating more pronounced depressive symptoms. The PHQ-9 demonstrated good internal consistency in this sample (Cronbach's *α* = 0.80).

The Generalized Anxiety Disorder Scale (GAD-7; Spitzer et al., 2006) was used to assess symptoms of generalized anxiety. Comprising of 7 items, this scale captures the key aspects of GAD. Participants can answer all items on a four-point Likert scale ranging from 0 (not at all) to 3 (nearly every day). The total scores range from 0 to 21 with higher scores indicating more severe symptoms of generalized anxiety. The GAD-7 has good psychometric properties (Spitzer et al., 2006; Löwe et al., 2008; Rutter and Brown, 2017). Reliability analysis for this study indicated that the GAD-7 had good internal consistency (Cronbach's *α* = 0.84).

The Perceived Stress scale (PSS-10; Cohen et al., 1983) was used as a self-report measure for perceived stress. With good psychometric properties (Cohen et al., 1983; Lee, 2012), the PSS-10 consists of 10 items measured on a five-point Likert scale ranging from 0 (never) to 4 (very often). The total scores can vary from 0 to 40, with higher scores indicative of higher perceived stress. The internal consistency of the PSS was supported, with Cronbach's *α* = 0.82.

The Mental Health Quality of Life questionnaire (MHQoL; van Krugten et al., 2022) was employed to assess the quality of life. It consists of 7 items and participants provide responses to each item on a four-point Likert scale ranging from 0 (very dissatisfied) to 3 (very satisfied). The aggregated scores can range from 0 to 21 with higher scores indicating higher quality of life. Research findings have substantiated that the MHQoL has commendable psychometric properties (van Krugten et al., 2022). The MHQOL scale demonstrated acceptable internal consistency (Cronbach's *α* = 0.77).

### 3.3 Moderators and covariates

Past research shows that the effects of internet-based interventions are heterogeneous (Ebert et al., 2013). Symptom severity and age have previously moderated the effectiveness of treatments (Karyotaki et al., 2018; Reins et al., 2021). Therefore, we evaluated various socio-demographic factors such as age, gender, ethnicity, marital status, study level, and whether participants are undergoing any form of psychotherapy or medication as potential covariates and moderators in our study.

### 3.4 Additional exploratory outcomes

Satisfaction with the intervention was measured with the Client Satisfaction Questionnaire (CSQ-8; Larsen et al., 1979). The scale consists of eight items on a four-point Likert scale ranging from 1 (*low satisfaction*) to 4 (*high satisfaction*). The total scores range from 8 to 32, with higher scores indicating greater satisfaction. The CSQ-8 has shown high reliability and validity for web-based interventions (Boß et al., 2016). With Cronbach's *α* = 0.90, the CSQ-8 showed very strong internal consistency in this sample.

The usability of the treatment was assessed by the System Usability Scale (SUS; Brooke, 1996). With good psychometric properties (Lewis, 2018) the SUS-10 consists of ten items measured on a five-point Likert scale from 1 (*strongly disagree*) to 5 (*strongly agree*). The total scores range from 0 to 100 with higher scores indicating higher usability. For this sample, the SUS indicated high internal consistency (Cronbach's *α* = 0.89).

Adherence to the program was defined as “the extent to which the user followed the program as it was originally designed” (Donkin et al., 2011). It was calculated by dividing the number of main modules completed by a participant at the time of the post-test by the total number of main modules in the program, and multiplying this by 100 (Etzelmueller et al., 2020). The resulting percentages serve as an indicator of the completion rate with higher percentages reflecting a higher level of treatment adherence. The adherence rates are reported separately for all participants and for those who completed at least one module.

The participants' satisfaction with the e-coach on treatment outcomes was assessed by the Working Alliance Inventory for guided internet interventions (WAI—I; Gómez Penedo et al., 2020). With adequate psychometric properties (Gómez Penedo et al., 2020), the WAI-I consists of twelve items on a five-point Likert scale with a total score ranging from 12 to 60, where higher scores indicate higher satisfaction. With Cronbach's *α* = 0.90, the WAI-I demonstrated excellent internal consistency in this sample.

### 3.5 Data analyses

All statistical analyses were conducted using STATA version 17. All individuals who were randomly assigned to the groups were included in the primary analyses according to the intent to treat principle (ITT). Missing values were imputed by multiple imputations with 100 imputed datasets using multivariate normal regression with an iterative Markov chain Monte Carlo (MCMC) method. A mixed-models linear regression was conducted with a restricted maximum likelihood algorithm for normally distributed data. In addition, a per-protocol analysis was performed, encompassing only those participants who successfully completed the post treatment and follow-up analyses.

To account for potential selective attrition, an additional sensitivity and robustness analysis was performed. While the ITT approach typically assumes data is Missing at Random (MAR), Cornelisz et al. (2020) argue that Missing Not at Random (MNAR) is a more appropriate assumption from a causal inference perspective. Following their recommendations, we combine Leebounds (Lee, 2009) with a Random Forest approach to estimate an effect interval in which the true unbiased treatment effect resides.

Changes in procrastination scores, depressive symptoms, anxiety symptoms, perceived stress and quality of life were assessed at post-treatment and follow-up assessments. The post-treatment score was the dependent variable, and the trial arm condition (intervention vs waitlist control) was the independent variable while adjusting for baseline scores. We calculated the effect size (Cohen's d) by subtracting the intervention group's average score on each measure from the average scores of the control group at the post-treatment and dividing the results by the pooled standard deviations of the respective measure. In addition, we calculated the means and standard deviations of the groups at all time points. For within-group effects, Cohen's d was calculated for the difference between baseline and 6-month follow-up scores in the intervention group. Finally, the secondary effects of the treatment on depression and anxiety were analysed separately for students scoring above the PHQ-9 (10 or more points) and GAD-7 (10 or more points) at baseline.

Clinically significant change in procrastination scores was determined by the reliability change index (RCI; Jacobson and Truax, 1991). The RCI was calculated using the baseline IPS scores, post-test IPS scores, the standard deviation of the baseline IPS scores and the interrater reliability of the IPS. The resulting RCI value indicated the reliability of observed change. An RCI less than −1.96 was classified as reliable improvement and an RCI greater than 1.96 as reliable deterioration.

Program/treatment experience-related information measured on the CSQ-8, SUS-10, WAI-I and adherence were evaluated descriptively. The means on the scales reflected participants' overall satisfaction with the program, alliance with the e-coaches and usability of the intervention. The adherence rate was calculated for all the participants randomized to the intervention group as well as for participants who completed at least one module (to reflect adherence for program starters).

## 4 Results

### 4.1 Demographic information

**Fig. 1:**
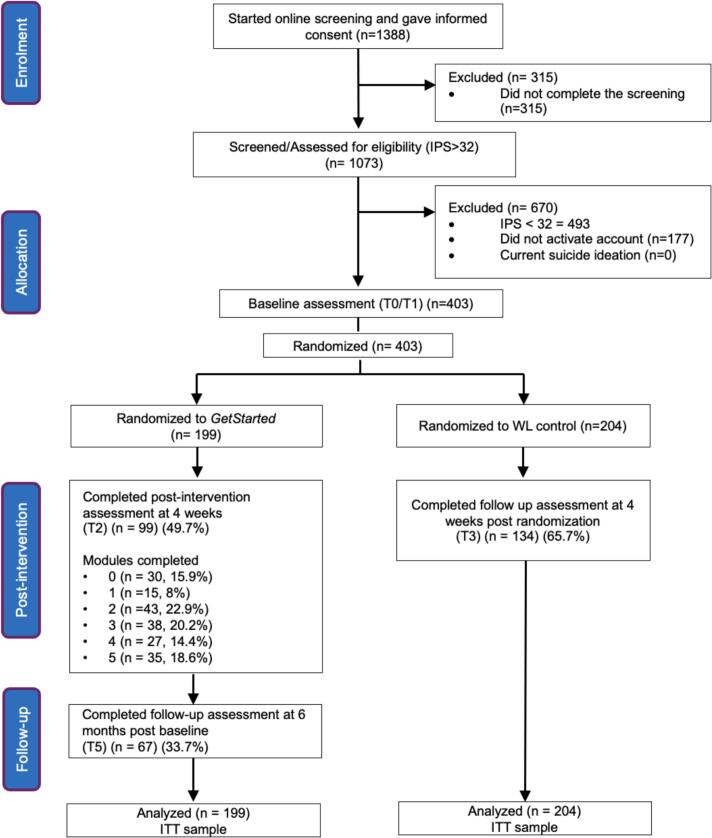
Participant flow diagram.

A total of 403 students were enrolled in the study and randomized to the intervention (*n* = 199) and waitlist control group (*n =* 204). See Fig. 1 for details on the randomization process and participant flow through the stages of this study. The mean age was 23.5 years (*SD =* 4.6). The majority were female (*n =* 281, 69.7 %), Dutch (*n =* 257, 63.7 %), single (*n =* 256, 63.5 %) and bachelor students (*n =* 221, 54.8 %). In total, 40 participants (9.9 %) were currently receiving psychological treatment, 34 participants (8.4 %) were on medications for psychological problems, and 18 participants (4.5 %) were both receiving psychological treatment and on medication for psychological problems. The average score on the IPS was 37.63 (*SD =* 3.34). All demographic information is presented in Table 1. There were no significant differences between the groups on any demographic characteristics. In addition, there were no significant pre-treatment differences between the intervention and control group on any of the primary or secondary outcome measures (*ps* > 0.05).

**Table 1:** Socio demographic characteristics of the participants and baseline differences between the groups (*n* = 403).

	Total (n = 403)	Intervention (*n* = 199)	Control (*n* = 204)	t / X^2^	*p*
Age	23.46 (4.62)	23.80 (5.06)	23.12 (4.12)	−1.49	0.136
Gender				3.10	0.212
Male	119 (29.53)	58 (14.39)	61 (15.14)		
Female	281 (69.73)	138 (34.24)	143 (35.48)		
Other	3 (0.74)	3 (0.74)	0		
Study level				1.07	0.586
Bachelor	221 (54.84)	104 (25.81)	117 (29.03)		
Master	169 (41.94)	88 (21.84)	81 (20.10)		
PhD	13 (3.23)	7 (1.74	6 (1.49)		
Nationality				0.04	0.851
International	146 (36.23)	73 (18.11)	73 (18.11)		
Dutch	257 (63.77)	126 (31.27)	131 (32.51)		
Marital status				1.19	0.754
Single	256 (63.52)	130 (32.26)	126 (31.27)		
In a relationship	129 (32.01)	62 (15.38)	67 (16.63)		
Married	12 (2.98)	5 (1.24)	7 (1.74)		
Other	6 (1.49)	2 (0.50)	4 (0.99)		
Current medical status				1.71	0.635
On medication	34 (8.44)	20 (4.96)	14 (3.47)		
Psychotherapy	40 (9.93)	21 (5.21)	19 (4.71)		
Both	18 (4.47)	8 (1.99)	10 (2.48)		
Baseline scores					
IPS	37.64 (3.34)	37.69 (3.40)	37.58 (3.28)	−0.35	0.730
PHQ-9	11.40 (5.13)	11.67 (4.98)	11.13 (5.26)	−1.07	0.285
GAD-7	9.08 (4.63)	9.35 (4.44)	8.81 (4.80)	−1.18	0.240

Note: Abbreviations (alphabetical): GAD-7: The Generalized Anxiety Disorder scale; IPS: The Irrational Procrastination Scale; PHQ-9: The Patient Health Questionnaire; p: significance at <0.05; t: t-test for continuous variables; X2: Pearsons's chi square for categorical variables.

### 4.2 ITT sample

The results of the LMM, along with the means and standard deviations on the IPS of the treatment and waitlist group, at baseline and at post-treatment (4 weeks post-baseline), are presented in Table 2, Table 3. There was a significant decrease in procrastination in the treatment compared to the waitlist control group at 4 weeks post baseline (*B =* −2.18, *SE =* 0.61, 95 % CI [−3.39, −0.98], *p <* .001) with a small - moderate effect size of Cohen's *d =* 0.38. In addition, a comparatively significant decrease in perceived stress (*B =* −2.23, *SE =* 0.69, 95 % CI [−3.59, −0.88], *p* < .002, *d =* 0.23) was noted as well. While there was an overall decrease in mean scores on the PHQ-9 & GAD-7 and an increase in MHQoL in the treatment group, these results were not statistically significant.

**Table 2:** Primary treatment effects GetStarted on procrastination.

Measure and condition	Pre		Post				
	n	Mean (SD)	n	Mean (SD)	B; SE (95 %CI)	Cohen's *d*	*p*
ITT sample							
*IPS*					−2.18; 0.61 (−3.38, −0.98)	0.38 (0.19, 0.58)	<0.001
Intervention	199	37.69 (3.4)	199	33.77 (5.4)			
Control	204	37.58 (3.3)	204	35.80 (5.2))			
Completers only							
*IPS*					−2.03; 0.54 (−3.08, −0.98)	0.44 (0.21, 0.73)	<0.001
Intervention	199	37.69 (3.4)	99	33.54 (5.2)			
Control	204	37.58 (3.3)	134	35.75 (4.3)			

Note: Abbreviations (alphabetical): B: unstandardised coefficient; CI: 95 % Confidence Interval; IPS: The Irrational Procrastination Scale; ITT: Intent to treat; n: total number of participants; *p*: significance at <0.05; SD: Standard deviation; SE: Standard error.

**Table 3:** Secondary treatment effects GetStarted.

Measure and condition	Pre		Post				
	n	Mean (SD)	n	Mean (SD)	B; SE (CI)	Cohen's *d*	*p*
ITT sample							
*PHQ9*					−0.60; 0.60 (−1.79, 0.58)	0.05 (−0.15, 0.24)	0.316
Intervention	199	11.67 (4.9)	199	9.14 (6.3)			
Control	204	11.13 (5.3)	204	9.41 (6.2)			
*GAD7*					−0.76; 0.52 (−1.78, 0.27)	0.07 (−0.13, 0.26)	0.146
Intervention	199	9.35 (4.4)	199	7.80 (6.4)			
Control	204	8.81 (4.8)	204	8.20 (5.9)			
*PSS10*					−2.23; 0.69 (−3.59, −0.88)	0.23 (0.04, 0.43)	0.002
Intervention	199	23.26 (5.7)	199	20.25 (7.5)			
Control	204	22.46 (6.2)	204	21.96 (7.2)			
*MHQOL*					0.65; 0.35 (−0.04, 1.35)	0.002 (−0.19, 0.20)	0.065
Intervention	199	11.73 (3.2)	199	12.89 (4.6)			
Control	204	12.13 (3.1)	204	12.90 (3.8)			


Completers only	n	Mean (SD)	n	Mean (SD)	B; SE (CI)	Cohen's *d*	*p*
*PHQ9*					−0.53; 0.56 (−1.62, 0.56)	0.11 (−0.16, 0.37)	0.344
Intervention	199	11.67 (4.9)	97	8.86 (4.9)			
Control	204	11.13 (5.3)	134	9.40 (5.2)			
*GAD7*					−0.86; 0.46 (−1.77, 0.05)	0.15 (−0.11, 0.42)	0.063
Intervention	199	9.35 (4.44)	96	7.41 (4.5)			
Control	204	8.81 (4.80)	134	8.16 (5.1)			
*PSS10*					−2.06; 0.62 (−3.29, −0.84)	0.32 (0.06, 0.59)	0.001
Intervention	199	23.26 (5.7)	97	20.07 (5.9)			
Control	204	22.46 (6.2)	134	22.07 (6.4)			
*MHQOL*					0.73; 0.33 (0.10, 1.37)	−0.14 (−0.4, 0.12)	0.024
Intervention	199	11.73 (3.2)	96	12.99 (3.0)			
Control	204	12.13 (3.1)	134	12.53 (3.5)			


Moderate to severe depression⁎	n	Mean (SD)	n	Mean (SD)	B; SE (CI)	Cohen's *d*	*p*
*PHQ9*					−0.46; 0.7 (−1.98, 1.07)	0.18 (−0.16, 0.51)	0.557
Intervention	127	14.57 (3.7)	60	10.63 (4.7)			
Control	118	14.65 (3.8)	79	11.48 (4.9)			
Moderate to severe anxiety⁎					−1.40; 0.8 (−3.00, 0.21)	0.57 (0.16, 0.99)	0.088
*GAD7*							
Intervention	95	13.13 (2.8)	42	9.74 (4.72)			
Control	78	13.93 (2.9)	51	12.35 (4.43)			

Note: Abbreviations (alphabetical): B: unstandardised coefficient; CI: 95 % Confidence Interval; GAD-7: The Generalized Anxiety Disorder scale; IPS: The Irrational Procrastination Scale; ITT: Intent to treat; MHQOL: The Mental Health Quality of Life questionnaire; n: total number of participants; *p*: significance at <0.05; PHQ-9: The Patient Health Questionnaire; PSS10 – The Perceived Stress Scale SD: Standard deviation; SE: Standard error.

⁎ Participants scoring 10 or more on the PHQ9 and GAD7 respectively.

The Random Forest Lee bounds procedure estimated a 95 % CI for the unbiased treatment effect ranging from −4.84 to 0.58. While the lower bound was statistically significant (*B =* −3.50, *SE =* 0.82, 95 % CI [−5.10, −1.90], *p* < .001), the upper bound was not. This conservative estimate suggests that the true unbiased effect likely reflects a reduction in symptoms, as most values in the confidence interval are negative. Overall, this finding supports the interpretation of a beneficial effect of the intervention.

### 4.3 Completers only

A total of 99 (49.7 %) participants out of the 199 randomized to the intervention group, completed the assessment at 4-weeks post-test. The results were similar and comparable to the ITT analyses (see Table 3). There were no baseline demographic differences between those who completed the assessments at post-test and those who did not. However, the completers had slightly lower baseline score for procrastination (*M* difference = 1.06, *p* = .028) and depressive symptoms (mean difference = 1.72, *p* = .014), indicating that participants who completed the post-test had marginally lower symptom severity at the start of the study.

### 4.4 Reliable change (improvement and deterioration)

Out of the 199 participants in the intervention group, 100 participants (50 %) had an RCI < −1.96. This means that half of the participants in the intervention group showed a reliable and significant improvement at the 95 % criterion. In comparison, 57 (28 %) participants out of the 204 randomized to the waitlist control group had an RCI < −1.96. The relative risk (RR) of significant improvement in the intervention group compared to the control group was 1.8, indicating that the participants in the intervention group were nearly twice as likely to show improvement in procrastination compared to the participants in the control group. Regarding deterioration, 14 participants (7 %) in the intervention group and 14 participants (7 %) in the control group had an RCI > 1.96, indicating reliable deterioration. The relative risk (RR) of deterioration in the intervention group compared to the control group was 0.99, suggesting no difference in risk of deterioration between groups.

### 4.5 Secondary treatment effects on moderate to severe depression and anxiety

In total, 245 participants scored 10 or more on the PHQ-9 at baseline, classifying them as having moderate to severe depressive symptoms. Of these, 139 participants completed the post-treatment assessment (*n* = 60 in the intervention group; *n* = 79 in the control group). Although there was a considerable decrease in the post-treatment mean scores on the PHQ-9, the effect of *GetStarted* was not statistically significant. The results were similar for the participants who scored 10 or more on the GAD-7 at baseline (*n =* 173), with a complete case sample of 93 participants (*n* = 42 in the intervention; *n* = 51 in the control group; see Table 3).

### 4.6 6-month follow-up

**Fig. 2:**
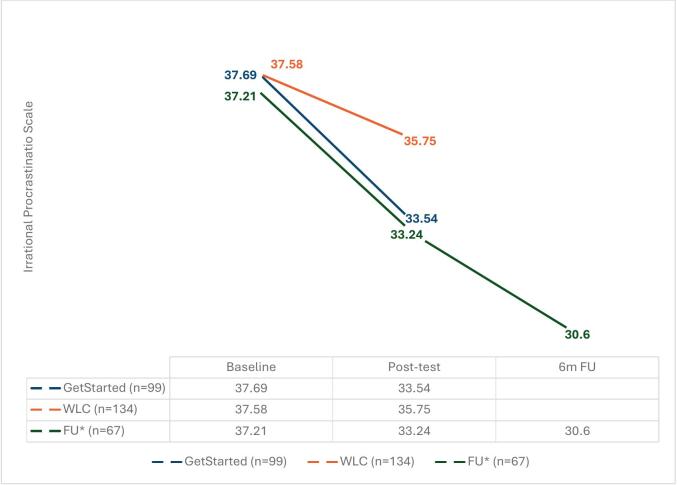
Mean scores on the Irrational Procrastination scale across the assessment points Note: *participants who completed the follow up measure at 6 months post baseline 95 % CI of pos*t*-test: *Getstarted*: (32.50, 34.67); WLC: (35.01, 36.49); FU: (31.72, 34.75) 95 % CI of 6-month follow up: FU: (29.00, 32.19).

The follow up assessment was completed by 67 participants from the intervention group (33.7 %). The mean scores indicated that procrastination remained significantly lower in the 6-month follow-up compared to baseline (*M* difference = 6.61; *SE =* 0.73; *p <* .001). In addition, depression, anxiety, and stress scores were also significantly reduced, and there was a corresponding increase in perceived quality of life (see Table 4). An important factor was that there were no significant baseline differences between these 67 participants and those who only completed the post-test measure. Therefore, it is possible that the 6-month follow-up scores for those who did not complete the measure would be similar to these 67 participants. The mean scores on the IPS across all the time point measures are displayed comparatively between both groups in Fig. 2.

**Table 4:** Within group⁎ mean difference from baseline to 6 month follow up (*N* = 67⁎⁎).

Measure	Pre	Post-test	Follow up		t-test⁎⁎⁎	
	Mean (SD)	Mean (SD)	Mean (SD)	t	Cohen's d (95 %CI)	p
IPS	37.21 (3.2)	33.24 (5.6)	30.60 (6.5)	9.05	1.11 (0.81, 1.41)	<0.000
PHQ9	11.31 (4.9)	8.72 (4.8)	7.82 (5.7)	5.63	0.69 (0.42, 0.95)	<0.001
GAD7	9.18 (4.2)	7.11 (4.3)	7.48 (5.1)	3.45	0.42 (0.17, 0.67)	0.001
PSS	22.75 (5.8)	19.80 (5.7)	18.45 (7.3)	5.37	0.66 (0.40, 0.93)	<0.001
MHQOL	11.66 (2.7)	13.33 (2.9)	13.30 (3.6)	−4.33	0.53 (0.28, 0.79)	<0.001

Note: Abbreviations (alphabetical): GAD-7: The Generalized Anxiety Disorder scale; IPS: The Irrational Procrastination Scale; MHQOL: The Mental Health Quality of Life questionnaire; N: total number of participants; *p*: significance at <0.05; PHQ-9: The Patient Health Questionnaire; PSS10 – The Perceived Stress Scale SD: Standard deviation.

⁎ Intervention group only.

⁎⁎ Number of participants who completed the follow up measure.

⁎⁎⁎ Within group difference in mean scores between baseline and follow up.

### 4.7 Program-related factors

Overall, the participants conveyed an excellent satisfaction with the usability of the intervention; 68 % scored above 85 on the SUS (SUS *M* *=* 85.77; *SD =* 5.2; Bangor et al., 2009). Similarly, 69 % scored above 24 on the CSQ-8 (CSQ-8 *M* *=* 24.9; *SD =* 3.8), indicating very good satisfaction with overall treatment. Satisfaction with the e-coaches was also high with a mean score of 46.23 (*SD =* 6.8) on the WAII, and 70.5 % of the participants scoring above 45. On average, the participants assigned to the intervention group completed 2.7 modules out of the 5 main modules. *E*-coaches spent approximately 20 min per module, amounting to an estimated 54 min of coach time per participant. The overall adherence rate was at 53 % for all participants in the intervention group and at 63 % for the participants who completed at least one module (see Table 5).

**Table 5:** Program related effect on treatment outcome.

Measures	N	Mean (SD)	B; SE (CI)	*p*
SUS	95	85.77 (5.2)	−0.15; 0.12 (−0.388, 0.080)	0.194
CSQ8	96	24.9 (3.8)	−0.49; 0.22 (−0.919, −0.057)	0.027
WAII	95	46.23 (6.8).	0.13; 0.13 (−0.123, 0.376)	0.318
Adherence⁎	100	63 %	0.003; 0.02 (−0.038, 0.046)	0.865

Note: Abbreviations (alphabetical): B: unstandardised coefficient; CI: 95 % Confidence Interval; CSQ8: Client Satisfaction Questionnaire; N: total number of participants; *p*: significance at <0.05; SD: Standard deviation; SE: Standard error; SUS: System Usability Scale; WAII: The Working Alliance Inventory for guided internet interventions.

⁎ Calculated for participants who completed at least 1 module.

Interestingly, only overall treatment satisfaction (CSQ-8) was significantly associated with treatment outcome (*B* = −0.49, *SE* = 0.22, 95 % CI [−0.92, −0.06], *p* < .05). There were no significant associations between treatment adherence, system usability, and satisfaction with e-coaches and post-test procrastination scores (see Table 5).

### 4.8 Safety monitoring

No adverse events or crisis situations were reported by participants or identified by e-coaches during the course of the study.

## 5 Discussion

The current study aimed to assess the effectiveness of an e-coach-guided internet-based intervention to overcome procrastination among university students. The results of our analyses indicated that *GetStarted* was significantly more effective in reducing procrastination compared to the waitlist control group with over 50 % of the participants in the intervention group reaching clinically relevant improvement. Yielding a moderate between-group effect size at post treatment, our findings are in line with previous RCTs that have tested the effectiveness of internet-based interventions for procrastination (Jeoung et al., 2023; Mutter et al., 2023; Rozental et al., 2015b, Rozental et al., 2018b). To the best of our knowledge, this is the first randomized controlled trial to examine the effectiveness of an internet-based intervention guided by trained clinical psychology student e-coaches for procrastination in a student population.

This study also examined the effects of *GetStarted* on secondary mental health outcomes such as depression, anxiety, stress, and quality of life. Previous studies have indicated that internet-based interventions targeting one specific psychosocial problem can also potentially reduce other common mental health complaints (Cuijpers, 2021; Grieve et al., 2021; Jeoung et al., 2023; Wade et al., 2019; Yang et al., 2022;). In our trial, however, only stress showed a statistically significant reduction. Depression, anxiety, and quality of life improved from baseline to post-test, but these changes did not differ significantly from the control condition - a pattern similar to findings from a guided iCBT for perfectionism (Shafran et al., 2017). Rozental et al. (2022) reported that severe procrastination co-occurred with higher depression, anxiety, and stress and lower quality of life, with negative impacts across multiple life domains. Our findings suggest that while the cross-sectional links with depression, anxiety, and quality of life may not translate into immediate improvements following a procrastination-focused intervention, reductions in stress could occur more rapidly. This is consistent with the idea that procrastination contributes directly to acute stress through last-minute work pressure, and alleviating these behaviours may relieve stress before broader mental health or life satisfaction benefits emerge. The absence of significant effects on depression, anxiety, and quality of life, highlight their possible indirect association with procrastination, the potential need for longer follow-ups to detect changes, and the possibility that this study was underpowered to detect small indirect effects on these outcomes.

An interesting finding was the difference in IPS scores from baseline to the 6-month follow up, which indicated that the treatment effect in the intervention group remained stable over a long-term period. Depression, anxiety, stress and quality of life were all significantly better at the 6-month follow-up compared to baseline. A total of 67 participants from the intervention group completed the follow up measure. Importantly, there were no significant baseline differences between those who completed the follow up measure and those who only completed the post-test measure. While the baseline similarity suggests that the 6-month follow up outcomes could be comparable to those who only completed the post-test measures, this assumption should be made with caution. Attrition bias cannot be ruled out as unmeasured factors influencing participants' decisions not to continue the follow up could also affect their outcomes. Future studies should consider exploring differences between completers and non-completers to better understand long term intervention effects. In addition, the 6-month follow-up could only be assessed in a within-group manner, a comparison to those who did not receive the intervention cannot be made. There are very few studies that have evaluated the long-term effects of an internet-based intervention for procrastination (Rozental et al., 2017; Van Eerde and Klingsieck, 2018). Therefore, our results add to the developing body of literature highlighting the long-term effects of internet-based interventions for procrastination.

Our results also indicated that the participants were highly satisfied with the intervention and had a very good user experience. The adherence rate was reasonable as well given the self-help nature of the intervention and the target population. Participants were also highly satisfied with their e-coaches, indicating that guidance by clinicians in training can be an effective form of high-quality support that can also overcome existing barriers such as the limited availability of psychologists and counsellors (Watkins et al., 2012). Overall, our results indicate a good acceptability of the *GetStarted* program among college students. Interestingly, treatment adherence was not significantly associated with treatment outcome. This finding may suggest that even partial engagement with the program, such as a single session (Schleider et al., 2025), may have been sufficient to yield benefits. However, further exploration is warranted to identify which components may be most effective. In addition, system usability and satisfaction with e-coaches were not significantly associated with treatment outcome suggesting that usability and supportive contact may enhance user experience but may not be sufficient to drive behaviour change.

No adverse events were reported, and reliable deterioration occurred in about 7 % of participants in both groups, with no between-group difference, suggesting that a small proportion of participants may experience worsening regardless of treatment allocation. However, this rate of deterioration can be considered low (Rozental et al., 2018b) and the absence of a group difference indicates that the intervention did not increase the risk of harm compared to a waitlist control.

Lastly, there were no significant differences in the effectiveness of treatment for different demographic groups indicating that *GetStarted* might be equally effective for reducing procrastination among all college/university students. However, larger trials with sufficient power will be more informative on this.

### 5.1 Strengths and limitations

This study has multiple strengths. First, it was adequately powered to detect a moderate between-group differences. Second, its use of an inactive comparator, allows us to more precisely distinguish the effect of the intervention. Third, the intervention was tested in the same setting where it will be implemented. This allows us to ensure its relevance, feasibility and effectiveness in real-world practice and supports the translation of our findings into meaningful improvements in practice. Fourth, the assessment of the participants' satisfaction with treatment, e-coaches, program usability and treatment adherence enabled us to identify areas of potential improvement that could increase adherence and improve treatment adherence. In addition, the personalized feedback on assignments and progress and a platform to communicate with an e-coach are promising approaches to allow students to recognize a problem and facilitate treatment-seeking behaviours (Czyz et al., 2013). Lastly, our multiple outcome measures and the follow-up assessment at 6-months allowed for a more nuanced understanding of the intervention effects.

However, these results should be interpreted in light of several limitations. First, despite our efforts with email reminders, motivation from e-coaches and phone call reminders, we still had a substantial number of participants who did not complete the treatment and post-test assessment. This indicates the need to further investigate strategies to improve adherence and reduce attrition. Second, while a waitlist control allowed us to establish preliminary efficacy, this design does not rule out non-specific influences such as expectancy or engagement (Goldberg et al., 2023). In addition, treatment effects may be overestimated when comparing a treatment to an inactive waitlist control (Furukawa et al., 2014). Therefore, different comparative designs (e.g., active controls) may be needed in future phases to clarify mechanisms of change. Third, long-term comparison between the treatment and waitlist control group was not possible as the control group had access to the program four weeks post-randomization. However, the 6-month follow-up measure allowed for a long-term within-group comparison for sustained treatment effectiveness. Fourth, we did not evaluate the influence of e-coach characteristics (e.g., sex, age, education) on treatment outcomes. Our primary aim was to assess the effectiveness of the intervention itself rather than coach-related effects, and in line with this, satisfaction with e-coaches was not associated with treatment outcomes. Nevertheless, future research could explore whether specific coach characteristics contribute to variability in outcomes. Finally, the questionnaires administered were self-report measures that are designed to reflect the self-assessments of actual behaviours. However, the responses could be influenced by factors such as willingness to disclose difficulties or overestimating them (Steel et al., 2001).

### 5.2 Directions for future research

A natural continuation of this study would be to evaluate whether similar effects could be achieved using a *GetStarted*-based single-session intervention. This approach is promising but requires testing (Schleider and Beidas, 2022). A single-session intervention could improve efficiency by reducing resources required (e.g., e-coaches will need less time) and could also overcome existing barriers such as time constraints experienced by students and lack of motivation students face when engaging in lengthy iCBT treatment programs (Rozental et al., 2015b). Another important direction would be to thoroughly assess the reasons for dropout and implement effective strategies to improve adherence. In addition, larger trials that are sufficiently powered to detect secondary treatment effects would provide more insight into the transdiagnostic benefits of specialized interventions such as *GetStarted* on common mental health problems. Moreover, incorporating ecological momentary assessments could help determine the chronology of change. Finally, although no adverse events and relatively low cases of deterioration occurred in this study, we acknowledge the importance of systematically monitoring potential harms in psychological intervention trials. Future research would benefit from incorporating open ended questions regarding negative experiences and unwanted effects (Rozental et al., 2015a; Rozental et al., 2018c). This would strengthen the evidence base for digital mental health interventions by capturing both efficacy and safety experiences.

## 6 Conclusions

*GetStarted* provides an effective, flexible and low intensity treatment for reducing procrastination among university and the effects are maintained at the 6-month follow up mark. Future studies should address dropout, adherence and potential secondary treatments effects on common mental health problems such as depression, anxiety and stress.

## Funding

The Caring Universities Project is funded by the 10.13039/501100001833Vrije Universiteit Amsterdam, 10.13039/501100001717Leiden University, 10.13039/501100001835Maastricht University, 10.13039/501100001829Utrecht University, 10.13039/501100001828Erasmus University Rotterdam, 10.13039/501100001827University of Amsterdam, Inholland University of Applied Sciences, Rotterdam University of Applied Sciences, and Avans University of Applied Sciences.

## Declaration of competing interest

The authors declare that they have no financial interest or personal relationships that could have appeared to influence the work reported in this paper.

## Appendix A Supplementary data

Supplementary material Image 1
