# Supplementary material for: Effectiveness of a guided internet-based intervention in reducing procrastination among university students – a randomized controlled trial
Source: Internet Interv. 2025 Sep 29;42:100878. doi: 10.1016/j.invent.2025.100878 (PMC12512977; doi:10.1016/j.invent.2025.100878)
Supplement: Supplementary file 1 — Supplementary material [file mmc1.docx]

APPENDIX

Caring Universities Consortium Scientific Committee Members

The following are the members of the scientific committee of the Caring Universities Consortium, listed by their affiliated institution. This committee consists of one or more senior researchers from each academic consortium partner:

- Vrije Universiteit Amsterdam:
 Sascha Struijs

- Leiden University:
 Nadia Garnesfski, Vivian Kraaij

- Utrecht University:
 Elske Salemink

- Maastricht University:
 Petra Hurks

- Erasmus University Rotterdam:
 Marilisa Boffo, Danielle Remmerswaal

- University of Amsterdam:
 Reinout Wiers, Claudia van der Heijde

- InHolland University of Applied Sciences:
 Lisa Klinkenberg

- Rotterdam University of Applied Sciences:
 Monique de Bruijn-Smolders

- Avans University of Applied Sciences:
 Jessica Nooij
